# Investigating the utility of the COM‐B and TM model to explain changes in eating behaviour during pregnancy: A longitudinal cohort study

**DOI:** 10.1111/bjhp.12590

**Published:** 2022-03-17

**Authors:** Lauren Rockliffe, Sarah Peters, Debbie M. Smith, Calvin Heal, Alexander E. P. Heazell

**Affiliations:** ^1^ Faculty of Biology, Medicine and Health Manchester Centre for Health Psychology School of Health Sciences University of Manchester UK; ^2^ Faculty of Biology, Medicine and Health Centre for Biostatistics University of Manchester UK; ^3^ Faculty of Biology, Medicine and Health, Maternal and Fetal Health Research Centre School of Medical Sciences University of Manchester UK; ^4^ St. Mary's Hospital, Central Manchester University Hospitals NHS Foundation Trust Manchester Academic Health Science Centre UK

**Keywords:** diet, eating, longitudinal studies, pregnancy, psychological theory, surveys and questionnaires

## Abstract

**Objectives:**

Pregnancy has been described as a ‘teachable moment’ for behaviour change, which presents an important opportunity for health promotion within antenatal care settings. However, no pregnancy‐specific model has been developed or tested in the context of health behaviour change during pregnancy. This study aimed to investigate and compare the utility of the Capability‐Opportunity‐Motivation Behaviour (COM‐B) and Teachable Moments (TM) models, to explain health behaviour change during pregnancy, within the context of eating behaviour.

**Design:**

Longitudinal cohort study.

**Methods:**

Five hundred and sixteen women completed a survey at between 12–16 weeks gestation (T1). Follow‐up data were collected at 20–24 weeks (T2), 36–40 weeks (T3), and 6–12 weeks postnatally (T4). The primary outcome was eating behaviour. To assess the utility of the COM‐B model, perceived capability, opportunity, and motivation to eat healthily were measured. To assess the utility of the TM model, risk perceptions, self‐image, and affective response were measured.

**Results:**

Overall, the COM‐B model explained 18.4% of the variance in eating behaviour, whilst the TM model explained 9%. Both models explained the most variance in eating behaviour at T1 and T3, compared with T2 and T4. Small changes were observed in eating behaviour and the model constructs over the time period studied, although these were not clinically meaningful.

**Conclusions:**

Neither the COM‐B nor TM model provide a satisfactory explanation of eating behaviour during pregnancy, however the findings suggest that certain stages of pregnancy may create more salient opportunities for behaviour change. The findings also support claims that motivation may not play a key role in directing eating behaviour during pregnancy. Further research is needed to explore the role of timing in antenatal behaviour change. The development of a pregnancy‐specific model is necessary to optimise understanding of pregnancy as a teachable moment for behaviour change.

**Statement of contribution**

***What is already known on this subject?***

- Pregnancy is regarded as a ‘teachable moment’ for health behaviour change.
- Existing models used to understand pregnancy as a teachable moment have not been developed, nor widely tested, in a pregnant population.
- The development of a pregnancy‐specific model of behaviour change is necessary.

***What does this study add?***

- The COM‐B and TM models provide an insufficient explanation of eating behaviour during pregnancy.
- Certain stages of pregnancy may provide more salient opportunities for behaviour change than others.
- Motivation may not play a key role in directing eating behaviour during pregnancy.

## Background

Pregnancy has been suggested to be an opportune time for health professionals to support women to initiate, or maintain, healthy behaviours. Increased contact with health professionals during this time and increased receptivity to health messages means that pregnancy is often described as a ‘teachable moment’ (Phelan, 2010). That is, a significant health event that motivates individuals to adopt risk‐reducing health behaviours (McBride, Emmons, & Lipkus, 2003). Lifestyle changes made during pregnancy have implications for long‐term health outcomes of both the mother and child, as the potential exists for newly initiated healthy behaviours to be maintained after birth.

McBride et al. (2003) first attempted to conceptualize the teachable moment within the context of smoking cessation. Their model (referred to hereon as the TM model) comprises three main psychological constructs underlying a health event, thought to act upon an individual’s motivation to change their health behaviour, their self‐efficacy, and acquisition of skills. These include an increased emotional or affective response, a change in self‐concept or social role, and increased perceived risk and outcome expectancies. This model provides a compelling argument for conceptualizing pregnancy as a teachable moment, as women are likely to experience heightened emotion in relation to their pregnancy, a redefining of social role and self‐concept as they prepare for the transition to motherhood, and an increase in perceived risk related to the health of the unborn child and to themselves (Phelan, 2010).

However, Olander, Darwin, Atkinson, Smith, and Gardner (2016) argue that the Capability–Opportunity–Motivation Behaviour model (COM‐B; Michie, van Stralen, & West, 2011) may offer an enhanced understanding of pregnancy as a teachable moment. The COM‐B model suggests that behaviour change has three necessary determinants: physical and psychological capability, physical and social opportunity, and reflective and automatic motivation. Within the context of pregnancy, these determinants are suggested to more usefully, and insightfully, explain behaviour change by moving beyond the traditional model of motivation, and considering that changes in capability and opportunity may also create opportune moments for intervention (Olander et al., 2016).

Whilst both the COM‐B and TM models appear to explain pregnancy as a teachable moment, neither has been tested prospectively in a population of pregnant women. Given that both models are used to explain antenatal behaviour change (e.g. eating behaviour), it is important to understand the efficacy of these models within this context. Further to this, pregnancy is often conceptualized as one teachable moment in and of itself (e.g. Atkinson, Shaw, & French, 2016; Phelan, 2010). However, it has been suggested that multiple teachable moments may occur throughout pregnancy, triggered by individual significant events, such as receiving confirmation of the pregnancy or attending the initial booking appointment, or more broadly related to different gestational trimesters (Olander et al., 2016). It is therefore important to consider the full spectrum of opportunities that pregnancy presents for health behaviour change and investigate how well existing behaviour change models account for these.

Making healthy changes to diet and eating behaviour during pregnancy is a key priority for many women (Maher & Lowe, 2015). Guidelines from the National Institute of Health and Care Excellence (NICE, 2021) recommend that health professionals discuss diet and nutrition with women from the first antenatal contact onwards, providing additional support for women with raised BMIs (NICE, 2010). Thus, utilizing this teachable moment to support women to initiate, or maintain, these healthy changes is of utmost importance. Not least because of the numerous poor maternal and foetal outcomes associated with poor diet or excessive weight gain during pregnancy (Kominiarek et al., 2018; Poston et al., 2016; Yang et al., 2019). In 2017, 21.6% of women in England were recorded as having a BMI of 30 or over at their booking appointment, which poses an increasing challenge for maternity and neonatal service provision (Public Health England, 2019). This far exceeds the prevalence of other risky health behaviours, such as smoking (10.4% at the time of delivery; NHS Digital, 2020), making it a significant public health priority. Understanding the extent to which existing models explain antenatal health behaviour, and identifying opportunities for behaviour change throughout pregnancy and the immediate postnatal period, will facilitate the development of more targeted interventions to improve maternal and infant health.

The purpose of this study was to investigate and compare the utility of the COM‐B model and TM model to explain health behaviour change during pregnancy, within the context of eating behaviour. The aims of the study were to:

1. Describe how the constructs of the COM‐B model, the constructs of the TM model, and eating behaviour change over time.
2. Investigate whether the COM‐B model or TM model better explains eating behaviour during pregnancy.
3. Examine whether certain time‐points throughout pregnancy act as more salient teachable moments than others.

## Methods

### Study design

This study is a longitudinal, prospective cohort study exploring changes in eating behaviour during pregnancy.

### Setting

Recruitment took place between February and June 2020. Shortly after commencing recruitment, it was announced that pregnant women were a high‐risk group for contracting COVID‐19 (16/03/2020; Department of Health & Social Care & Hancock, 2020) and the first national lockdown was enforced in England (23/03/2020–23/06/2020). Two further lockdowns were subsequently imposed (05/11/2020–02/12/2020, and 06/01/2021–08/03/2021). Data collection was ongoing until February 2021. From hereon, the term ‘lockdown’ will refer to any point after 16/03/2020.

### Sample and recruitment

Women were recruited using both active and passive recruitment methods. Active recruitment took place in seven NHS maternity units in the North‐West of England. Research midwives approached women face‐to‐face to invite them to participate and maternity units also advertised the study using flyers and posters, where appropriate. Passive recruitment involved advertising the study online using various channels, including paid adverts (e.g. Facebook, Instagram), social media/forum posts (e.g. Twitter, Reddit, MumsNet), university mailing lists, and recruitment websites.

Women were eligible to participate if they were over the age of 18 and between 12 and 16 weeks pregnant at the time of recruitment. Women also needed to be receiving maternity care in the United Kingdom, with a singleton pregnancy, and able to read and write in English to participate. Women unable to provide informed consent were not eligible to participate.

As an incentive, participants were offered the opportunity to be entered into a prize draw to win a £100 shopping voucher, for each of the four surveys they completed. Women were provided with necessary details about study participation, but no additional information on the topic under investigation was provided.

### Data collection

Data were collected at four time‐points: between 12 and 16 weeks of pregnancy (T1), 20–24 weeks (T2), 36–40 weeks (T3), and 6–12 weeks postnatally (T4). Informed consent was gained at each time‐point. T1 surveys were completed online using Research Electronic Data Capture (REDCap) tools hosted at the sponsoring university (Harris et al., 2009, 2019), or in a paper format. All follow‐up surveys were completed online. Data collected in maternity settings included the use of both online and paper surveys.

Participant contact details and their expected due date were collected at T1 to allow for further follow‐up and for the dates of the follow‐up surveys to be plotted. Participants were sent a link to the follow‐up surveys either by email and/or text message, depending on the contact details provided, and were sent a reminder after 2 weeks for the antenatal surveys and after 4 weeks for the postnatal survey.

Favourable ethical opinion was granted by the Health Research Authority (HRA) and the NHS Research Ethics Committee (REC) (IRAS ID: 264741; REC Reference: 19/NW/0674).

### Survey measures

Unless specified, items were generated for the present study. Details of the measures used are presented in Table 1.

**Table 1:** Survey measures

Variable	Measure	Description
Demographic and medical characteristics	Items were created for the survey or based on response items from the 2011 UK census (Office for National Statistics, 2011)	N/A
Nausea and/or vomiting frequency	The Pregnancy Unique‐Quantification of Emesis scoring system (PUQE; Koren et al., 2005)	The PUQE was used to measure severity of nausea and vomiting in the study sample, as it was hypothesized that these symptoms may affect participants’ eating behaviour Participants were asked to rate their physical symptoms using a 5‐point response scale. These ratings were summed to create a composite score indicating no symptoms (0–3), mild (4–6), moderate (7–12), or severe symptoms (≥13)
Perceived risk	Items were based on a measure developed by McBride, Blocklin, Lipkus, Klein, and Brandon (2017)	Participants were asked to respond to four statements assessing their level of concern about their health and that of their baby during their pregnancy, using a 7‐point scale (ranging from “strongly disagree” to “strongly agree”) Two composite scores were created using pro‐rated averages (perceived risk to self and perceived risk to baby)
Self‐image		Participants were asked to rate how they felt about becoming a mother on a 7‐point scale (ranging from “negative” to “positive”), to select one of three statements that best reflected how they currently felt about themselves (“I feel better about myself”, “I feel worse about myself”, “there has been no change in how I feel about myself”), and to rate on a 7‐point scale (ranging from “strongly disagree” to “strongly agree”) their agreement with the statement *“most people important to me think I will be a good mother”* A composite score was created using pro‐rated averages (self‐image).
Worry		Participants were asked to respond to two statements assessing levels of worry about their health and that of their baby during their pregnancy, using a 5‐point scale (ranging from “not worried at all” to “very worried”) A composite score was created using pro‐rated averages (worry).
Positive and negative affect	The Positive and Negative Affect Scale (PANAS; Watson, Clark, & Tellegen, 1988)	The PANAS was used to measure positive and negative affect, as it was originally used alongside the measure developed by McBride et al. (2017) Participants were asked to rate the extent to which they had experienced 20 emotions in the past few weeks using a 5‐point scale (ranging from “very slightly or not at all” to “extremely”) Two composite scores were created using pro‐rated averages (positive affect and negative affect)
COM‐B constructs	Items were based on a measure developed by Taylor et al. (2016)	Participants were asked to rate their agreement with 18 statements relating to their perceived capability to eat healthily, (e.g. “I find it easy to eat healthily”), perceived opportunity, (e.g. “It is easy for me to eat healthily at home and at work”), and motivation (e.g. “I have healthy eating goals that I want to achieve”), using a 7‐point scale (ranging from “strongly disagree” to “strongly agree”) Composite scores were created for each of the model constructs using pro‐rated averages
Eating behaviour	The Short Form Food Frequency Questionnaire (SFFFQ; Cleghorn et al., 2016)	The SFFFQ was selected to as an appropriate measure of dietary quality as it was developed in a UK adult population including women of reproductive age. Whilst the measure does not account for the specific dietary recommendations of pregnant or breastfeeding women, broad food groups are used to assess dietary quality (e.g. ‘fruit’, ‘beans or pulses’, ‘fibre‐rich breakfast cereal’) which does not limit reporting Participants were asked to report the frequency with which they had eaten various food items during a “typical” week over the previous month, using an 8‐point scale (ranging from “rarely or never” to “5 + a day”) and a 6‐point scale (ranging from “rarely or never” to “7+ times a week”), to rate 13 food items and seven food items, respectively These scores were combined to create a single dietary quality score ranging from 5 to 15, which indicated optimum dietary intake of these foods. A healthy diet was defined as having an overall dietary quality score of >12, as stated in the original measure

### Analysis

Data were analysed using IBM SPSS Statistics, version 25. Data were cleaned and composite scores created, where <20% data were missing, for all model constructs and the PUQE items. Frequencies and descriptive statistics were generated to assess sample characteristics and mean scores at each time‐point for all available data. Independent *t*‐tests and chi‐squared tests were conducted to compare variable means at T1 for different demographic groups, to further explore the data and check for differences between groups. Mixed regression models were performed to compare changes in scores over time in order to make use of all available data at each time‐point. Multiple linear regression models were used to identify which model better explains eating behaviour, controlling for time, age, ethnicity, BMI, education, IMD decile, parity, gestational diabetes, prior pregnancy complications, experience of sickness/nausea, and pre/post‐lockdown recruitment. Controlling for the same potential confounders (without time), individual regression models built for each time‐point were then used to assess the associations between model constructs and dietary quality, to identify if certain time‐points act as more salient teachable moments than others during pregnancy. Experience of sickness/nausea and perceived risk to baby were not included for the T4 analysis as these were not measured at this time‐point. Adjusted *R* ^2^ is reported for all regression analyses.

## Results

Five hundred and sixteen participants completed the survey at the first time‐point (T1). Two hundred and sixteen (41.9%) participants were recruited online and 300 (58.1%) via NHS maternity services. Three hundred and two (58.5%) participants participated using the online survey and 214 (41.5%) using the paper version. Most participants (*n* = 302, 58.5%) were recruited prior to lockdown.

**Figure 1:**
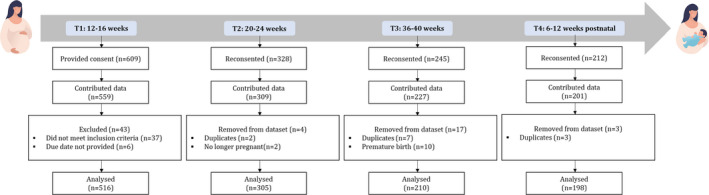
Data collection process.

Participant attrition was relatively high, with 305 (59.1%), 210 (40.7%), and 198 (38.4%) participants providing data at T2, T3, and T4, respectively (see Figure 1 for an overview of the data collection process). One hundred and forty‐four participants provided data at all four time‐points (27.9%). Twelve participants withdrew from the study after completing the T1 survey, four at T2, eight at T3, and two at T4.

### Recruitment

41.5% (*n* = 214) of participants were recruited post‐lockdown. Compared to those recruited pre‐lockdown, these participants perceived greater risk to their own health (mean difference 0.67 (95% CI, 0.94–0.39), *p* < .001) and to the health of their baby (mean difference 0.72 (95% CI, 1.03–0.41), *p *< .001). Greater levels of worry about their health and that of their baby (mean difference 0.46 (95% CI, 0.63–0.29), *p* < .001), as well as greater perceived capability to eat healthily (mean difference 0.33 (95% CI, 0.55–0.11), *p* = .004) were also reported.

Participants who were recruited online (*n* = 216, 41.9%) reported greater risk perceptions about their own health (mean difference 0.86 (95% CI, 0.59–1.14), *p *< .001) and that of their baby (mean difference 0.96 (95% CI, 0.66–1.27), *p* < .001), higher levels of worry about their health and that of the baby (mean difference 0.52 (95% CI, 0.35–0.69), *p* < .001), and higher levels of negative affect (mean difference 0.17 (95% CI, 0.04–0.29), *p* = .009) than those recruited in maternity care settings. This is congruent with the above findings relating to lockdown recruitment, as the majority of participants completing online surveys were also recruited post‐lockdown.

### Missing data

For the composite scores of key variables, the amount of missing data was low, with 2.8% missing at T1, 4% at T2, 3% at T3, and 6.5% at T4, for those who completed the survey. There was a general trend in missing data increasing slightly as participants progressed through the surveys, but overall, the spread of missing data was fairly even across variables at each time‐point and there did not appear to be a pattern of missingness.

At T1, participants who provided data at all four time‐points (*n* = 144, 27.9%) reported higher levels of perceived risk about their own health during pregnancy (mean difference 0.31 (95% CI, 0.62–0.00), *p* = .049) and greater levels of worry about their own health and that of their baby during pregnancy (mean difference 0.21 (95% CI, 0.40–0.02), *p* = .03) than participants who did not complete all four surveys. No association was found between survey completeness and age (*p* = .09), ethnicity (*p* = .72, Fisher’s Exact Test), or prior pregnancy difficulties or complications (*X* ^2^ (1) = 0.35, *p* = .62), and parity (*X* ^2^ (1) = 1.05, *p* = .33). However, participants who were educated to a postgraduate or higher level were more likely to provide data at all four time‐points (*p* = .03, Fisher’s Exact Test), as were those who were recruited online (*X* ^2^ (1) = 5.44, *p* = .02), and those from higher IMD quintiles (*X* ^2^ (4) = 11.47, *p* = .02).

### Sample characteristics

Participants were aged between 18 and 45 years (*M* = 30.1; *SD* = 4.8). The majority of participants were from White backgrounds (*n* = 474; 91.9%), married (*n* = 259, 50.2%), and in full‐time employment (*n* = 312, 60.5%). Most participants had obtained a higher or postgraduate education level qualification (*n* = 302, 58.5%), and more participants lived in the most deprived 10% of areas in England (*n* = 74, 14.3%) than in any other decile. At T1, 41.1% of participants had a healthy BMI (*n* = 212).

Participants had a mean gestational age of 13.5 weeks (range = 11–16 weeks, *SD* = 1.2), 20.7 weeks (range = 19–25 weeks, *SD* = 1.0), and 36.4 weeks (range = 36–40 weeks, *SD* = 0.9) at T1, T2, and T3, respectively, and a mean of 6.5 weeks (range = 2–12 weeks, *SD* = 1.6) postpartum at T4. The largest proportion of participants had no existing children (*n* = 249, 48.3%).

17.6% of participants reported experiencing difficulty conceiving prior to their current pregnancy (*n* = 91) and 5.0% (*n* = 26) had used assisted reproductive technology to conceive. These figures are broadly reflective of those reported in the general population (Human Fertilisation & Embryology Authority, 2021; National Institute for Health & Care Excellence, 2013; Office for National Statistics, 2020). However, 26.0% of participants had experienced at least one prior miscarriage (*n* = 134) and 1.4% had experienced a stillbirth (*n* = 7), which appears to be higher than general population rates (Office for National Statistics, 2021; Quenby et al., 2021). See Table 2 for sample characteristics.

**Table 2:** Sample characteristics at T1

Characteristics	All participants (*n* = 516)
Mean age in years (range, *SD*)	30 (18–45, 4.8)
Weeks pregnant/postpartum (range, *SD*)	
T1	13.5 weeks (11–16, 1.2)
T2	20.7 weeks (19–25, 1.0)
T3	36.4 weeks (36–40, 0.9)
T4	6.5 weeks postpartum (2–12, 1.6)
Number of children (%)	
0	249 (48.3%)
1	166 (32.2%)
2	72 (14.0%)
3+	23 (4.5%)
Missing	6 (1.2%)
Prior pregnancy difficulties (%)	
Difficulty conceiving	91 (17.6%)
Assisted pregnancy	26 (5.0%)
Miscarriage	134 (26.0%)
Stillbirth	7 (1.4%)
Ethnic group (%)	
White	474 (91.9%)
Mixed/Multiple ethnic groups	15 (2.9%)
Asian/Asian British	9 (1.7%)
Black/African/Caribbean/Black British	3 (0.6%)
Other ethnic groups	3 (0.6%)
Missing	12 (2.3%)
Marital status (%)	
Single	23 (4.5%)
In a relationship	226 (43.8%)
Married	259 (50.2%)
Separated	3 (0.6%)
Divorced	1 (0.2%)
Missing	4 (0.8%)
Employment status (%)	
Employed full‐time	312 (60.5%)
Employed part‐time	90 (17.4%)
Self‐employed full‐time	14 (2.7%)
Self‐employed part‐time	12 (2.3%)
Full‐time student	9 (1.7%)
Part‐time student	2 (0.4%)
Unemployed	63 (12.2%)
Other	11 (2.1%)
Missing	3 (0.6%)
Education level (%)	
Postgraduate education	130 (25.2%)
Higher education	172 (33.3%)
Further education	140 (27.1%)
High school	62 (12.0%)
No formal qualifications	9 (1.7%)
Other	1 (0.2%)
Missing	2 (0.4%)
Levels of neighbourhood deprivation^a^ (%)	
1 (most deprived 10%)	74 (14.3%)
2	41 (7.9%)
3	43 (8.3%)
4	40 (7.8%)
5	35 (6.8%)
6	37 (7.2%)
7	34 (6.6%)
8	37 (7.2%)
9	45 (8.7%)
10 (least deprived 10%)	39 (7.6%)
Missing	91 (17.6%)
BMI category at T1 (kg/m^2^)(%)^b^	
Severely obese (>40)	12 (2.3%)
Obese (30–39.9)	99 (19.2%)
Overweight (25–29.9)	152 (29.5%)
Healthy weight (18.5–24.9)	212 (41.1%)
Underweight (<18.4)	8 (1.6%)
Missing	33 (6.4%)

^a^ Based on the English Indices of Deprivation deciles (Ministry of Housing Communities & Local Government, 2019).

^b^ Based on NIHR guidance (National Institute for Health & Care Excellence, 2014).

### Pregnancy difficulties or complications

Participants who reported experiencing any type of prior pregnancy difficulties or complications (*n* = 204, 39.5%) reported higher levels of worry about their own health and that of their baby during their pregnancy (mean difference 0.19 (95% CI, 0.02–0.37), *p* = .03) than those participants who had not experienced this.

### Sickness and nausea

At T1, most participants reported experiencing mild (*n* = 225, 43.6%) or moderate (*n* = 134, 26%) symptoms of nausea and vomiting. At T2 and T3, the majority of participants reported experiencing no symptoms (T2: *n* = 204, 66.9%; T3: *n* = 126, 60%). See Table S1 for all scores across time‐points.

### Gestational diabetes

By T3, 7.6% (*n* = 16) of participants reported developing gestational diabetes. This is similar to rates of gestational diabetes reported in the general population of pregnant women in Europe (5.4%; Eades, Cameron, & Evans, 2017). On average, these participants reported higher dietary quality scores (mean difference 1.07 (95% CI, 0.12–2.01), *p* = .03) than participants without gestational diabetes at T3.

### Examining changes in model constructs and eating behaviour over time

#### Changes in COM‐B model constructs over time

Participants’ perceived capability to eat healthily was significantly lower at T4 than at T1 (mean difference −0.24 (95% CI, −0.46 to −0.02), *p* = .03), T2 (mean difference −0.37 (95% CI, −0.60 to −0.13), *p* < .001), and T3 (mean difference −0.35 (95% CI, −0.57 to −0.14) *p* < .001).

Similarly, perceived opportunity to eat healthily was significantly less at T4 than at T1 (mean difference −0.41 (95% CI, −0.59 to −0.23) *p *< .001), T2 (mean difference −0.44 (95% CI, −0.64 to −0.24) *p* < .001), and T3 (mean difference −0.30 (95% CI, −0.49 to −0.11), *p* < .001).

There were no statistically significant differences in participants’ motivation to eat healthily from T1 through to T4.

#### Changes in TM model constructs over time

There were no statistically significant differences in participants’ perceived risk to their own health from T1 through to T4. However, participants’ perceived risk towards the health of their baby was significantly higher at T2 than T1 (mean difference 0.25 (95% CI, 0.04–0.47), *p* = .01).

There were no statistically significant differences in participants’ self‐image from T1 through to T4.

Participants’ levels of worry about their health and that of the baby reduced significantly from T1 to T4 (mean difference −0.25 (95% CI, −0.44 to −0.07), *p* = .002), and from T2 to T4 (mean difference −0.28 (95% CI, −0.47 to −0.10), *p *< .001).

Positive affect significantly increased across time‐points. Levels of positive affect were significantly higher at T2 (mean difference 0.17 (95% CI, 0.07–0.27), *p* < .001), T3 (mean difference 0.15 (95% CI, 0.04–0.26), *p* = .002), and T4 (mean difference 0.34 (95% CI, 0.20–0.48), *p* < .001), than at T1. Positive affect at T4 was also significantly higher than at T2 (mean difference 0.17 (95% CI, 0.03–0.31), *p* = .01) and T3 (mean difference 0.19 (95% CI, 0.05–0.33), *p* = .002). There were no statistically significant differences in negative affect at different time‐points.

#### Changes in eating behaviour over time

After initially increasing from T1 (*M* = 9.68, *SD* = 1.98) to T2 (*M* = 9.97, *SD* = 2.02), dietary quality decreased over subsequent time‐points (T3: *M* = 9.82, *SD* = 1.86; T4: *M* = 9.55, *SD* = 1.86), with a significant decrease in quality from T2 to T4 (mean difference −0.39 (95% CI, −0.77 to −0.02), *p* = .03).

**Figure 2:**
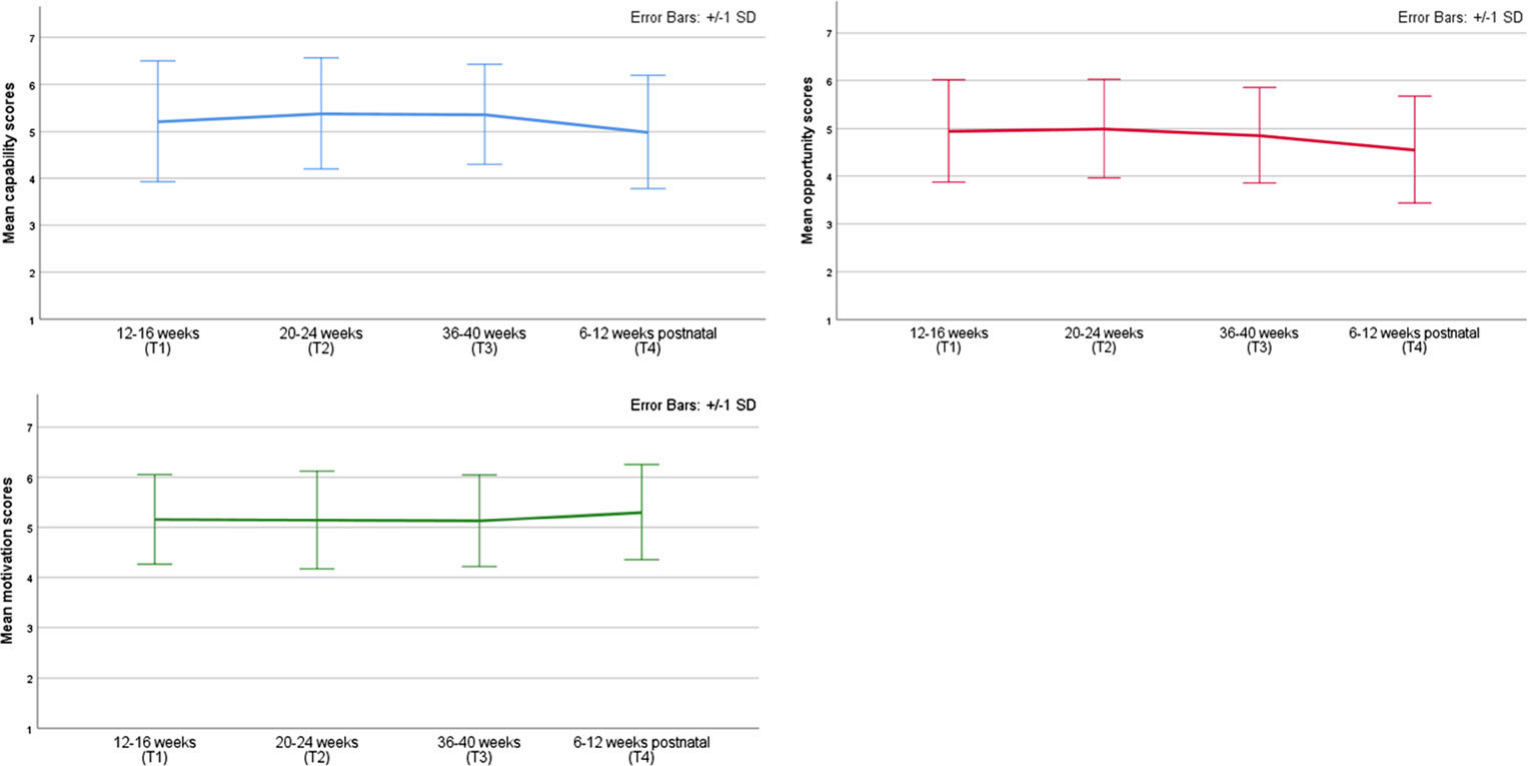
Mean scores for Capability, Opportunity, and Motivation across time‐points.

**Figure 3:**
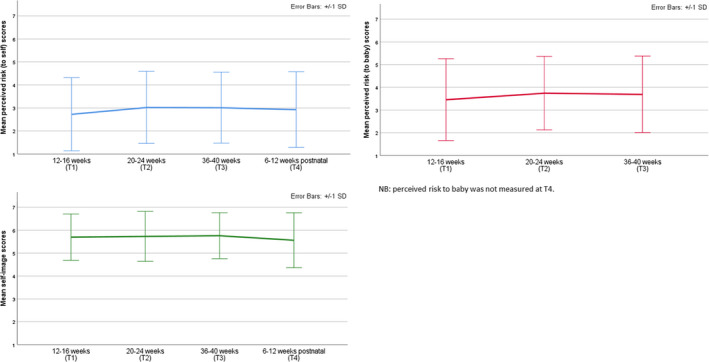
Mean scores for perceived risk to self, perceived risk to baby, and self‐image across time‐points.

**Figure 4:**
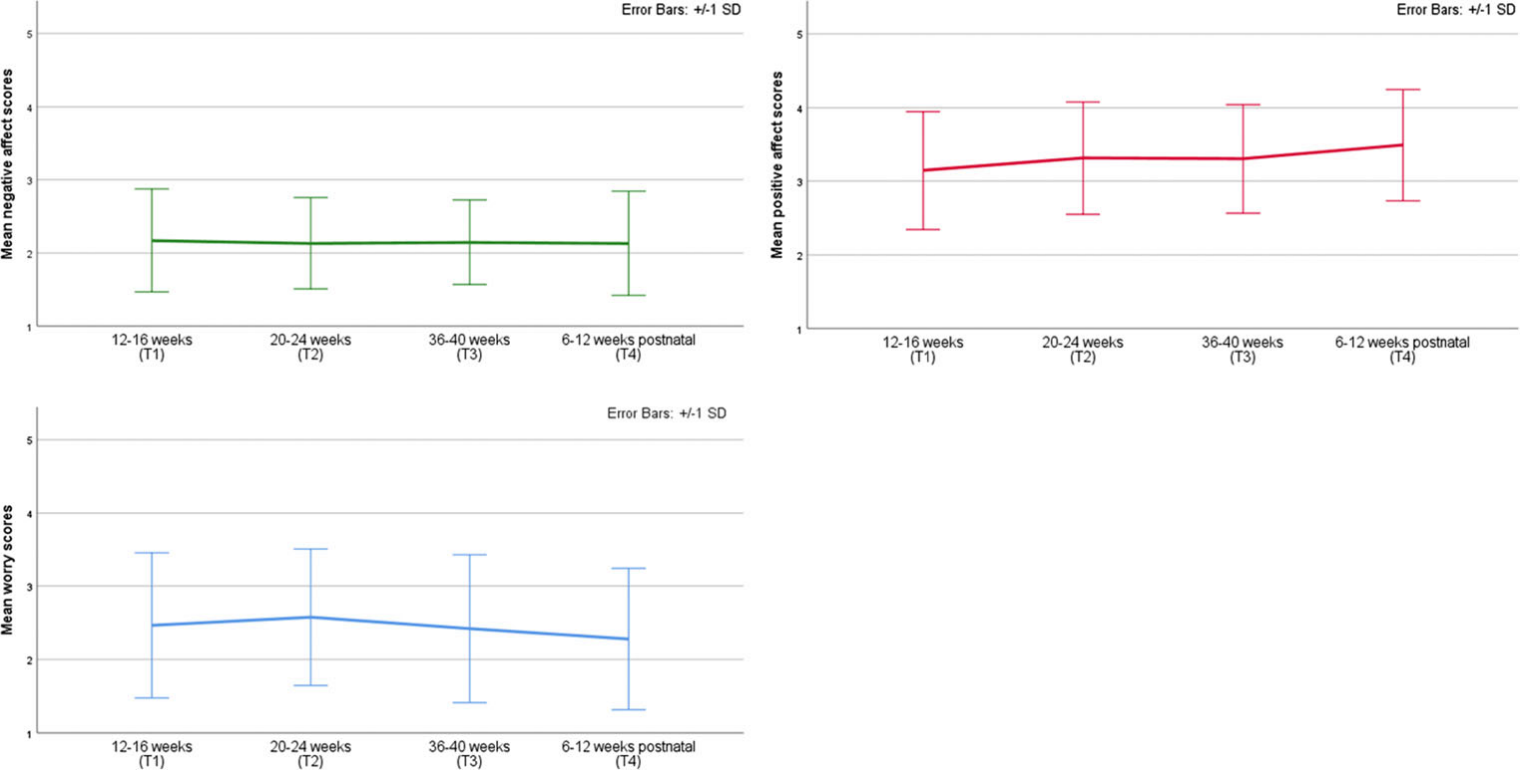
Mean worry, positive and negative affective scores across time‐points.

Mean scores for the COM‐B and TM model constructs and dietary quality at each time‐point are presented in Table 3 and in Figures 2, 3, 4.

**Table 3:** Mean scores for model constructs and dietary quality at each time‐point, for all available data

	12–16 weeks (T1)		20–24 weeks (T2)		36–40 weeks (T3)		6–12 weeks postnatal (T4)	
	*n* = 516		*n* = 305		*n* = 210		*n* = 198	
	*n*	*M* (*SD*)	*n*	*M* (*SD*)	*n*	*M* (*SD*)	*n*	*M* (*SD*)
COM‐B constructs								
Capability	494	5.21 (1.29)	292	5.38 (1.18)	202	5.36 (1.07)	183	4.99 (1.21)
Opportunity	497	4.94 (1.07)	292	4.99 (1.03)	202	4.85 (1.00)	184	4.55 (1.12)
Motivation	498	5.16 (0.89)	292	5.15 (0.97)	202	5.13 (0.91)	184	5.30 (0.95)
TM constructs								
Risk to self	509	2.73 (1.59)	293	3.02 (1.56)	205	3.01 (1.54)	187	2.93 (1.64)
Risk to baby	509	3.45 (1.80)	292	3.74 (1.62)	205	3.70 (1.68)	–	–
Self‐image	502	5.70 (1.01)	294	5.73 (1.09)	205	5.76 (1.00)	185	5.56 (1.20)
Worry	508	2.46 (0.99)	293	2.58 (0.93)	204	2.42 (1.01)	187	2.28 (0.96)
Positive affect	498	3.15 (0.80)	294	3.31 (0.76)	205	3.30 (0.74)	187	3.49 (0.76)
Negative affect	498	2.17 (0.70)	294	2.13 (0.63)	205	2.14 (0.58)	187	2.13 (0.71)
Dietary quality	500	9.68 (1.98)	291	9.97 (2.02)	202	9.82 (1.86)	183	9.55 (1.86)

### Investigating whether the TM model or COM‐B model better explains eating behaviour during pregnancy

#### Overall variance in eating behaviour explained by the COM‐B model

After including potential confounders, for every one unit increase in perceived capability score, the dietary quality score will on average increase by 0.30 (95% CI, 0.06–0.53, *p* = .01) and for every one unit increase in opportunity score, the dietary quality score will on average increase by 0.42 (95% CI, 0.11–0.73, *p* = .008). There was no statistically significant association between motivation and dietary quality. These constructs account for 18.4% of the overall variance in dietary quality when including confounding variables.

#### Overall variance in eating behaviour explained by the TM model

After including potential confounders, for every one unit increase in positive affect score, the dietary quality score will on average increase by 0.29 (95% CI, 0.02–0.56, *p* = .04). There was no statistically significant association between negative affect, worry, self‐image, or risk perception scores and dietary quality scores. These constructs account for 9% of the overall variance in dietary quality.

When excluding model constructs, the confounding factors accounted for 6.8% of the overall variance in dietary quality.

A summary of the regression coefficients is presented in Table 4.

**Table 4:** Summary of the regression analysis for model constructs predicting dietary quality

	*B*	95% CI	β	*p*	Adjusted *R* ^2^ including potential confounders	Adjusted *R* ^2^ without constructs
COM‐B constructs						
(Constant)	8.02	(4.81, 11.24)		<.001	.184	.068
Capability	0.30	(0.06, 0.53)	0.18	.01		
Opportunity	0.42	(0.11, 0.73)	0.20	.008		
Motivation	0.08	(−0.17, 0.33)	0.04	.54		
TM constructs						
(Constant)	10.69	(6.98, 14.40)		<.001	.090	.068
Risk to self	0.13	(−0.05, 0.31)	0.10	.14		
Risk to baby	−0.08	(−0.26, 0.09)	−0.07	.36		
Self‐image	0.13	(−0.09, 0.35)	0.07	.25		
Worry	0.08	(−0.24, 0.39)	0.04	.64		
Positive affect	0.29	(0.02, 0.56)	0.12	.04		
Negative affect	−0.35	(−0.73, 0.03)	−0.11	.07		

### Examining whether certain time‐points act as more salient teachable moments than others during pregnancy

#### Variance in eating behaviour explained by the COM‐B model at different time‐points

Across time‐points, the amount of variance in dietary quality explained by the COM‐B model constructs was fairly similar, with 19.2%, 13.8%, 18.6%, and 16.9% of the overall variance in dietary quality explained at T1, T2, T3, and T4, respectively. In line with the previous findings, there was a significant association between perceived capability (unstandardized B 0.58 (95% CI, 0.22–0.93) *p* = .002) and opportunity (unstandardized B 0.61 (95% CI, 0.07–1.14) *p* = .03), and dietary quality scores, at T1 and T3 respectively.

#### Variance in eating behaviour explained by the TM model at different time‐points

The TM model constructs account for 9.1%, 2.7%, 12.5%, and 4.5% of the overall variance in dietary quality at T1, T2, T3, and T4, respectively. At T2, a significant association was found between positive affect and dietary quality scores (unstandardized B 0.68 (95% CI, 0.11–1.25) *p* = .02). There were no other significant associations at any time‐point.

Although dietary quality scores remained relatively stable over time, the constructs of both models accounted for the most variability in dietary quality at T1 and T3. See Table 5 for a summary of the regression analysis for model constructs predicting dietary quality when adjusting for potential confounders at each time‐point.

**Table 5:** Summary of the regression analysis for model constructs predicting dietary quality when adjusting for potential confounders at each time‐point

	12–16 weeks (T1)			20–24 weeks (T2)			36–40 weeks (T3)			6–12 weeks postnatal (T4)		
	*B* (95% CI)	*p*	Adjusted *R* ^2^	*B* (95% CI)	*p*	Adjusted *R* ^2^	*B* (95% CI)	*p*	Adjusted *R* ^2^	*B* (95% CI)	*p*	Adjusted *R* ^2^
COM‐B constructs												
(Constant)	7.77 (2.95, 12.59)	.002	.192	5.56 (−1.36, 12.48)	.11	.138	8.51 (2.45, 14.57)	.006	.186	11.56 (5.20, 17.92)	.001	.169
Capability	0.58 (0.22, 0.93)	.002		−0.09 (−0.60, 0.42)	.73		0.16 (−0.31, 0.63)	.51		0.02 (−0.45, 0.49)	.92	
Opportunity	0.15 (−0.33, 0.64)	.54		0.71 (−0.01, 1.44)	.05		0.61 (0.07, 1.14)	.03		0.64 (−0.04, 1.32)	.07	
Motivation	0.09 (−0.33, 0.50)	.68		0.31 (−0.19, 0.81)	.22		−0.12 (−0.59, 0.35)	.61		0.06 (−0.40, 0.52)	.80	
TM constructs												
(Constant)	8.43 (2.17, 14.68)	.009	.091	10.91 (3.52, 18.29)	.004	.027	11.48 (4.33, 18.62)	.002	.125	13.20 (4.95, 21.46)	.002	.045
Risk to self	0.24 (−0.02, 0.51)	.07		−0.09 (−0.46, 0.28)	.62		0.17 (−0.18, 0.53)	.34		0.03 (−0.24, 0.30)	.80	
Risk to baby	0.12 (−0.16, 0.39)	.40		−0.07 (−0.43, 0.30)	.72		−0.23 (−0.56, 0.10)	.17		–	–	
Worry	−0.30 (−0.85, 0.25)	.28		0.13 (−0.50, 0.76)	.68		0.19 (−0.35, 0.72)	.49		0.01 (−0.50, 0.51)	.99	
Positive affect	0.25 (−0.16, 0.66)	.23		0.68 (0.11, 1.25)	.02		−0.12 (−0.62, 0.39)	.65		0.22 (−0.38, 0.81)	.47	
Negative affect	−0.29 (−0.88, 0.31)	.35		0.11 (−0.69, 0.91)	.79		−0.47 (−1.21, 0.27)	.21		−0.24 (−0.92, 0.45)	.50	
Self‐image	0.18 (−0.24, 0.59)	.40		−0.11 (−0.49, 0.27)	.56		0.33 (−0.08, 0.74)	.11		0.16 (−0.23, 0.55)	.42	

## Discussion

This study investigated the extent to which the COM‐B and TM models explain health behaviour change during pregnancy, within the context of eating behaviour. The results reveal small changes in eating behaviour and the model constructs throughout pregnancy and the early postnatal period. Both models explained the most variance in eating behaviour in early‐ and late‐pregnancy compared with mid‐pregnancy and the postnatal period. Overall, the COM‐B model explained more of the variance in eating behaviour than the TM model.

Whilst the COM‐B and TM models have been suggested to be useful models to understand pregnancy as a teachable moment, both assume that women’s behaviour will be influenced by simultaneous changes in the individual psychological constructs. The findings of this study revealed marginal changes in these individual constructs at different points throughout pregnancy; for example, women’s worries about their health and that of their baby decreased slightly over time, whilst levels of positive affect increased. Risk perceptions about the health of the baby also increased slightly from early‐ to mid‐pregnancy, and capability and opportunity were lowest in the postnatal period. Although these findings were statistically significant, the changes observed were arguably too small to be clinically meaningful. However, our findings suggest that there may be some fluidity in the extent to which these psychological constructs are experienced throughout pregnancy and the postnatal period. This is important as neither the COM‐B nor TM model consider that the salience of their respective constructs might change at different points in time. Consequently, previous research has suggested that these models may be limited when applied to the context of pregnancy, due to the changeable nature of the pregnancy experience and the static understanding of behaviour change that these models offer (Rockliffe, Peters, Heazell, & Smith, 2021a, 2021b). It will therefore be important to further explore nuances in the salience of the constructs over time, in future work.

Minimal changes in eating behaviour were also found throughout pregnancy, and whilst there was a statistically significant difference from T2 to T3, this did not reflect a meaningful change. Similar findings have been reported elsewhere in the literature which suggests that eating behaviour remains relatively stable throughout pregnancy (Crozier et al., 2009; Cucó et al., 2006). This may be as a result of advice received from health care professionals to maintain a healthy diet, in line with current clinical guidance in the United Kingdom (NICE, 2008; NHS, 2020). More marked changes have been reported in relation to other health behaviours, such as smoking and alcohol use (Crozier et al., 2009). Neither the COM‐B nor TM model were developed in the context of eating behaviour but were tested within this context as there is an important need to support women to make healthier changes in this area, as previously discussed. McBride et al. (2003) have also argued that focusing on a single behaviour in this way facilitates comparison across studies and enables testing to assess whether teachable moments exist for other behavioural outcomes. As such, it may be advantageous to explore the utility of the COM‐B and TM models to explain changes in other health behaviours, including those that are pregnancy specific (e.g. vitamin supplementation, adherence to dietary recommendations).

Overall, the COM‐B model explained more of the variance in eating behaviour than the TM model. This finding supports the supposition made by Olander et al. (2016) that the COM‐B model may offer a more meaningful explanation of pregnancy as a teachable moment by moving beyond the concept of motivation as the primary driver for behaviour change. This argument was supported in this study as an association was found between capability and opportunity and eating behaviour, but not between motivation and eating behaviour. This may also explain why associations were not found between many of the TM model constructs and eating behaviour, as to a large extent they reflect changes in both automatic and reflective motivation (Olander et al., 2016).

The COM‐B model explained 18.4% of the variance in eating behaviour. This finding is broadly consistent with other studies that have investigated the utility of behaviour change models to explain eating behaviour in various contexts (ranging from 3.4% to 23%) (Malek, Umberger, Makrides, & ShaoJia, 2017; McEachan, Conner, Taylor, & Lawton, 2011; Willmott, Pang, & Rundle‐Thiele, 2021). Nonetheless, our findings indicate that a large proportion of the variance in eating behaviour is explained by unmeasured factors, again demonstrating the inability of both the COM‐B and TM models to sufficiently explain eating behaviour during pregnancy. Further research is therefore necessary to develop a model of behaviour change that is specific to women’s pregnancy experience, in order that it can more fully explain and conceptualize the psychological mechanisms underpinning behaviour change at this time, not otherwise captured by existing models.

In thinking about the unexplained, or unmeasured, variance explaining eating behaviour, it is possible that certain facets of the COM‐B constructs may not have been captured using the study measures. For example, participants’ physical capability was assessed using items that asked about capability to prepare and eat healthy foods. However, pregnancy symptoms, such as sickness and nausea, often restrict women’s capability to eat (Crozier, Inskip, Godfrey, Cooper, & Robinson, 2017), which may not have been fully captured. Similarly, physical opportunity was measured using items asking about ease of healthy eating at home and work, for example. However, socioeconomic deprivation is a factor understood to restrict physical opportunity to eat healthily (Marmot, Allen, Boyce, Goldblatt, & Morrison, 2020), which will also not have been fully reflected in the data collected. Some of the variance unaccounted for by the COM‐B constructs may therefore still reflect capability, opportunity, and motivation, but require a more sensitive measure to effectively record these aspects. With regard to the TM model, previous research has suggested that when applied to pregnancy, the model fails to account for non‐psychological factors, such as practical and environmental factors, social influences, and physical pregnancy symptoms (Rockliffe, Peters, Heazell, & Smith, 2021b). These may therefore reflect some of the unmeasured factors contributing to eating behaviour that the TM model failed to include.

Another source of variance could be experience in prior pregnancy, as the current study sample included women with different parity. Whilst this was controlled for in the analysis, we acknowledge that this will not have removed all effects, as these may differ depending on the woman’s prior experience of pregnancy. As such, it may be valuable for future research to explore the utility of the COM‐B and TM models to explain eating behaviour separately in nulliparous and multiparous women, to identify potential differences that may have implications for the tailoring of theory and intervention design.

Both the COM‐B model and TM model were found to explain the most variance in eating behaviour in early‐ (12–16 weeks) and late‐pregnancy (36–40 weeks), compared with mid‐pregnancy (20–24 weeks) and the postnatal period (6–12 weeks postpartum). This suggests that there may be certain stages of pregnancy that present more effective opportunities, or teachable moments, for women to change their eating behaviour. These findings support claims made by Olander et al. (2016) who suggested that certain gestational stages, or antenatal events, may act as individual teachable moments. For example, receiving confirmation of the pregnancy, or attending initial antenatal appointments may help to encourage change (Olander et al., 2016), whereas first trimester nausea or sickness, or increased fatigue experienced in the last trimester may act as a barrier (Cheng, Chou, Wang, Tsai, & Liou, 2015; Rockliffe, Peters, Heazell, & Smith, 2021a).

These findings strengthen the argument for the development of a pregnancy‐specific model of behaviour change that considers the potential for certain stages of pregnancy to provide more opportune moments for change. However, it is evident that further research is needed to better understand the influence of timing on women’s health behaviour. Utilizing a qualitative approach may provide an enhanced understanding of the different factors influencing women’s eating behaviour at different time‐points throughout pregnancy, that goes beyond that captured using a quantitative approach. Furthermore, it is important to consider the different influences on women’s eating behaviour during the antenatal and postnatal period, and associated implications for the development of behavioural support during these times.

### Strengths and limitations

This is the first study to compare existing models of behaviour change in the context of antenatal eating behaviour. Longitudinal data were collected from a large sample of women throughout the duration of their pregnancies, providing new insight that has highlighted the insufficiency of existing models to explain eating behaviour during pregnancy. However, there are several limitations that must be considered.

Firstly, the vast majority of participants in the sample were from a White background. Whilst a high proportion of residents in England and Wales identify as White (86%; Office for National Statistics, 2015), the sample in this study exceeds this (91.9%). This therefore limits our ability to generalize the findings to women from non‐White backgrounds in England, which may be problematic given disparities in pregnancy outcomes experienced by women from Black and Asian ethnic backgrounds (MBRACE‐UK, 2020). A review of the literature identified areas of inequality affecting women from ethnic minority backgrounds which included communication issues and the relationship women had with their midwife (Khan, 2021). Receiving a lack of support or advice from health professionals may act as a barrier to behaviour change due to reduced social opportunity or psychological capability (COM‐B model), or lowered risk perceptions (TM model). An absence of data from these women may therefore mean that the data are biased, by not reflecting these experiences. It is therefore important to keep this in mind when interpreting the findings. Future work would benefit from taking a purposive recruitment approach in order to reach different ethnic groups.

A further limitation is that the study relied on the use of self‐report measures. Whilst this was the most appropriate method to collect longitudinal data from a large sample, it is important to acknowledge that self‐report questionnaires are susceptible to social desirability biases. It is possible that women under‐reported the amount, or types, of food they consumed in order to avoid perceived judgement. Similarly, women may have provided more positive responses to questions surrounding self‐image as a new mother, as it may be perceived to be socially unacceptable to admit that others do not believe you would be a good mother (Meeussen & Van Laar, 2018; Staneva, Bogossian, Morawska, & Wittkowski, 2017). In terms of the data collected, this would mean that it could potentially be skewed in an upward direction for these items, inflating the importance of positive self‐image, for example, on eating behaviour.

Participants were recruited around the time the first UK lockdown was enforced. Research conducted during lockdown reported heightened levels of perceived threat (Qi, Li, Liu, Li, & Huang, 2020) and elevated anxiety in pregnant women related, in part, to concern about threat to their lives and that of their baby (Lebel, MacKinnon, Bagshawe, Tomfohr‐Madsen, & Giesbrecht, 2020). Similar differences were found in our sample comparing T1 scores of those recruited before and during lockdown. Furthermore, changes in dietary intake have been reported during this time (Whitaker, Hung, Alberg, Hair, & Liu, 2021; Zhang et al., 2020) and recent research has shown that women who were pregnant during the pandemic reported higher levels of negative affect and lower levels of positive affect than women pregnant before the pandemic (Berthelot et al., 2020). To account for any differences in scores of those recruited pre‐ or post‐lockdown, this factor was included as a potential confounder in the main analysis. However, it is important to consider other impacts of the pandemic beyond those of lockdown, such as the role of obesity and diabetes in increased COVID‐19 disease severity (Holly, Biernacka, Maskell, & Perks, 2020). Over a fifth of participants in this study were categorized as obese or severely obese at T1, and a proportion of participants also reported being diagnosed with gestational diabetes at T3 (although these were consistent with prior reports in English and European populations respectively). As such, this may have contributed to increased risk perceptions relating to their own health or that of their baby.

### Implications for theory and clinical practice

The study findings revealed that the role of motivation in women’s eating behaviour was not as salient as the roles of capability and opportunity in directing behaviour, which is important given the onus often placed on motivation as a tool to change behaviour. In clinical practice, it may therefore be advantageous to provide support around capability and opportunity, in addition to motivation. For example, ensuring appropriate provision of information (psychological capability) or being mindful of practical barriers faced by women from socioeconomically deprived backgrounds (physical opportunity), such as high food costs, time constraints, or lack of availability of healthy food. From a clinical perspective, the findings highlight the importance of considering gestational stages when delivering interventions or health promotional advice. NICE guidelines (2021) recommend that information about diet and nutrition are provided at the first antenatal appointment, and that the delivery of information (not specific to diet and nutrition) should be tailored to the timing and stage of a woman’s pregnancy. Beyond this however, there is limited guidance provided about the provision of behaviour change advice, or about the influence of different gestational stages on a woman’s ability to change her behaviour. Going forward it will be important to develop a pregnancy‐specific model of behaviour change that can help to support health care professionals in delivering appropriately timed interventions that are sensitive to the unique physiological and psychological events occurring throughout the antenatal period.

### Conclusions

Whilst the COM‐B model explained more variance in eating behaviour during pregnancy than the TM model, neither model provides a sufficient explanation. However, both models explained more variance in eating behaviour in early‐ and late‐pregnancy than in mid‐pregnancy or the postnatal period, suggesting that certain gestational stages might afford more effective teachable moments. Furthermore, motivation may not play a key role in eating behaviour change. Further research is required to better understand the influence of timing in eating behaviour during pregnancy, and for the development of a pregnancy‐specific model of behaviour change.

## Funding

This study was funded by the Medical Research Council under Grant MR/N013751/1 and supported by the NIHR Clinical Research Network Greater Manchester. The views expressed are those of the authors and not necessarily those of the Medical Research Council, NHS, NIHR, or the Department of Health and Social Care. AEPH is funded by Tommy’s Charity via a grant to the Maternal and Fetal Health Research Centre.

## Conflicts of interest

All authors declare no conflict of interest.

## Supporting information


**Table S1**. Severity of sickness/nausea across time‐points.

## Data Availability

Data are available upon reasonable request.
