# Supplementary material for: Investigating the utility of the COM‐B and TM model to explain changes in eating behaviour during pregnancy: A longitudinal cohort study
Source: Br J Health Psychol. 2022 Mar 17;27(3):1077–99. doi: 10.1111/bjhp.12590 (PMC9541598; doi:10.1111/bjhp.12590)
Supplement: Supplementary file 1 — Table S1. Severity of sickness/nausea across time‐points. [file BJHP-27-1077-s001.docx]

**Supplementary material**

|  |  |  | **No symptoms** | **Mild** | **Moderate** | **Severe** | **Missing** |
| --- | --- | --- | --- | --- | --- | --- | --- |
|  | **n** | **M (SD)** | **n (%)** | **n (%)** | **n (%)** | **n (%)** | **n (%)** |
| **T1** | 514 | 5.3 (2.4) | 150 (29.1%) | 225 (43.6%) | 134 (26%) | 5 (1) | 2 (0.4%) |
| **T2** | 304 | 3.7 (1.5) | 204 (66.9%) | 87 (28.5%) | 12 (3.9%) | 1 (0.3%) | 1 (0.3%) |
| **T3** | 210 | 3.7 (1.4) | 126 (60%) | 78 (37.1%) | 4 (1.9%) | 2 (1%) | - |

Table S1. Severity of sickness/nausea across time-points
